# Food protein induced enterocolitis to fish in children

**DOI:** 10.1186/2045-7022-5-S3-P28

**Published:** 2015-03-30

**Authors:** Purificacion Gonzalez-Delgado, M Victoria Moreno, Fernando Clemente, Esther Caparros, Javier Fernandez

**Affiliations:** 1 Allergy Unit, General Hospital Alicante, Alicante, Spain; 2 Immunology Deparment, UMH, Alicante, Spain; 3 Pediatric Service, General Hospital Alicante, Alicante, Spain

## 

Food protein induced enterocolitis syndrome (FPIES) is a severe infantile form of cell-mediated, food hypersensitivity caused typically by cow`s milk and/or soy characterized by profuse vomiting and diarrhea, with progression to dehydration and shock in some cases. FPIES caused by fish is uncommon.

## Methods

Children admitted for evaluation of gastrointestinal symptoms after fish ingestion. Skin Prick Tets (SPT) with commercial extracts of codfish, hake, sole, tuna, herring, haddock were performed. Specific IgE to fish was also determined.

Patch test with the same extracts for SPT were applied 48h and read at 2 and 24 hours after removing. Open Oral challenge (OOC) was performed when diagnosis was not clearly established with clinical history.

## Results

We report 14 children with FPIES caused by fish. All had negative SPT to fish. Serum specific IgE was undetectable in all.

Median age at the diagnosis was 9.46 months (8-11).

Diagnosis was established after a median of 4 previous reactions (3-5).

Symptoms appeared 1-4 hours after intake (median 2h). 9 patients were treated in Emergency room because profuse vomiting, 3 were admitted in Pediatric Intensive Care Unit as they developed shock with hypotension. OOC was performed in 3 patients being positive.

Patch test were positive in 6/14 patients.

## Conclusions

We communicate 14 patients with FPIES induced by fish. Diagnosis of FPIES especially when triggered by fish is often delayed because of a low index of suspicion and clinical features that overlap with sepsis and with other diseases. Sensitivity of patch test was 42.8%.

